# Learning from Exemplars in Global Health: a road map for mitigating indirect effects of COVID-19 on maternal and child health

**DOI:** 10.1136/bmjgh-2020-003430

**Published:** 2020-07-31

**Authors:** David E Phillips, Zulfiqar A Bhutta, Agnes Binagwaho, Ties Boerma, Matthew C Freeman, Lisa R Hirschhorn, Raj Panjabi, On behalf of the Exemplars in Global Health Partnership, Abdoulaye Maiga

**Affiliations:** 1 Health and Life Sciences, Gates Ventures, Seattle, Washington, USA; 2 Centre for Global Child Health, The Hospital for Sick Children, Toronto, Ontario, Canada; 3 Office of the Vice Chancellor, University of Global Health Equity, Kigali, Rwanda; 4 Rady Faculty of Health Sciences, University of Manitoba, Winnipeg, Manitoba, Canada; 5 Rollins School of Public Health, Emory University, Atlanta, Georgia, USA; 6 Northwestern University Feinberg School of Medicine, Chicago, Illinois, USA; 7 Last Mile Health, Boston, Massachusetts, United States

**Keywords:** child health, maternal health, nutrition

**Summary box**

- To minimise these negative indirect effects, countries will need to consider all domains of health systems, including demand, supply, resources and social determinants.
- To this end, learning from countries that have improved health outcomes amid other crises could provide helpful strategies.
- Some of the strategies used by these positive outlier countries include clear national leadership, data-driven targeting, community-focused health services and a strong emphasis on equity.
- Studying positive outlier countries to find lessons applicable for other settings is the focus of the recently launched Exemplars in Global Health programme.

## Introduction - Indirect effects of COVID-19 threaten greater maternal and child health losses than direct effects

The effect of COVID-19 on health and health systems around the world has been unprecedented. Now with over 15 million confirmed cases and 618 000 confirmed deaths globally, the toll on human health has been a tragedy.^1^ Daily cases continue to increase as well; the 3-day rolling average of new confirmed cases was 246 597 on July 18, up from 223 597 one week prior.^2^ And while daily confirmed deaths have levelled off from their peak in April,^3^ hospital resource use is at risk of returning to levels that exceed intensive care unit capacity in 92 out of 160 countries with available projections in 2020.^4^

This excess resource use across health systems threatens to impact more patients than just those suffering from coronavirus disease. The strain it places on medical staff, supplies and facility capacity could cause enormous disruptions to primary healthcare in general, limiting the ability to adequately respond to and prevent infectious disease, non-communicable disease and injuries alike.

The policy and funding response to the COVID-19 pandemic, necessary as it is, creates further challenges than health system strain, however. Financial resources have already begun diverting away from routine services and interventions, as financial channels such as the Global Fund allow the reprogramming of funds originally intended for other purposes.^5^ Demand to use health services is also being interrupted as physical distancing remains an effective mitigation strategy and understandable concerns about disease transmission create hesitance to seek care.^6 7^ Worse, the broader economic impact of the pandemic, coupled with existing healthcare inequalities and a disproportionate disease burden, is exacerbating socioeconomic disparities and the social determinants of health.^8^

The combination of these four forces—constrained supply, reduced resources, suppressed demand and worsening socioeconomic inequality—creates a likelihood that the indirect effects on health and nutrition will be more harmful than the direct health consequences of the disease. Projections already suggest that COVID-19 could lead to up to a 45% increase in child deaths and a 39% increase in maternal deaths across low and middle-income countries.^1^ Such a backslide would undermine dramatic gains made since 2000. Under-five mortality has declined by 43% globally, with 4.3 million fewer annual deaths in 2017 than 2000.^9^ During the same period, the prevalence of stunted growth in children under age 5 declined from 32% to 22%, or 48.7 million fewer children.^10^

Progress in maternal and child health is at especially high risk during the COVID-19 response.^11^ The suspension of national immunisation programmes has been projected to cause 140 child deaths per COVID-19 death averted,^12^ putting 80 million infants at risk of contracting vaccine-preventable diseases.^13^ Disruptions in distribution campaigns for insecticide-treated bed nets and effective antimalarial treatments have been projected to set malaria mortality back to levels not seen in 20 years under the worst scenario, with most of those affected being children.^14^ The socioeconomic consequences of the pandemic could leave mothers and children especially vulnerable to health risks,^15^ and pose new challenges for the utilisation and access of healthcare.^8^

## The challenge of avoiding indirect effects on maternal and child health is immense, but not impossible

While the imperative to avoid indirect effects is clear, the solutions are less so. Much of the discussion surrounding these dire forecasts has been to reinforce the importance of avoiding indirect effects and the key areas that must not go unattended.^16–18^ A more focused approach on rapid learning, policy development, adapting, testing and scaling previously proven interventions and delivery strategies will be essential to preventing the potential consequences.

The challenge of navigating this complex crisis and avoiding indirect effects is significant. Countries must tend to all four of the domains mentioned above at once, including (1) supply: avoiding the closure of health facilities, faltering supply chains and potential health worker shortages, while maintaining the safety of all front-line worker; (2) resources: ensuring that neither the response to the epidemic nor subsequent economic decline diverts resources from maternal and child health and nutrition support services; (3) demand: safely averting a reduction in use of primary health services; and (4) social determinants: ensuring that declining socioeconomic circumstances do not exacerbate inequities and the social determinants of health.^15 16 18^

Some positive outlier countries have proven especially adept at improving health outcomes via the four domains indirectly threatened by the pandemic and response though, even amid other crises. Examples can be found around the world in countries that have achieved exceptional health gains against the odds. In this commentary, we examine the experiences of four such countries: Liberia, Rwanda, Peru and Bangladesh, whose key strategies to success align with these four domains (table 1).

**Table 1:** Country examples and strategies for success

Domain	Country example	Strategies for success
Supply	Community health workers (CHWs) in Liberia	Investment in salaries and supervision for CHWs Development of supply and information systems
Resources	Coordination of donors in Rwanda	Adherence to a clear, evidence-based national vision Integration of vertical programmes and decentralisation
Demand	Demand-generation programmes in Peru	Programmes to support insurance coverage, conditional cash transfer and results-based financing Data-driven approach to equity
Social determinants	Women’s empowerment in Bangladesh	Utilisation of local NGO sector Microfinancing and education programmes targeting women

CHW, community health worker; NGO, non-governmental organisation.

## Part of the solution to avoiding indirect effects of COVID-19 may be to learn lessons from positive outliers

Learning what these and other countries did—and how they did it despite limited resources—could offer important lessons about how to replicate their successes in today’s context. Many of these best-performing countries employed strategies for delivering interventions developed during past emergencies, and structured programmes to achieve the above health gains despite limited resources. Their experiences offer lessons in each of the aforementioned four domains and they often found ways to address multiple issues at once.

Liberia bolstered its supply side with a remarkable community health worker (CHW) programme. Civil war left the country with only 51 physicians as of 2005, and an Ebola epidemic less than a decade later claimed the lives of 8% of its health workers and infected over 10 000 people. In response, Liberia’s CHWs played a vital part in reducing transmission by promoting social distancing and contact tracing, detecting and referring individuals with suspected Ebola virus disease (EVD) for testing, and encouraging patients with EVD to seek care early.^19^ In 2015, as the Ebola epidemic was brought under control, Liberia enacted bold reforms. First, it doubled the number of CHWs to 21 per 10 000 people to reach remote rural communities. Liberia also invested in systems to support these workers: contracting and paying them, supervising them with nurses, building a supply chain to deliver medicines to them across over 3000 remote rural communities and leveraging digital platforms to train them and create a robust information system.^20^ Liberia’s CHWs carried out over 3.3 million door-to-door home visits, and the country has leveraged the same systems to train, equip and support its CHWs to help prevent the spread of COVID-19, screen for COVID-19 symptoms and carry out contact tracing for contacts of those infected with COVID-19.^21 22^

Rwanda has often been seen as one of the world’s hallmark examples of child mortality reduction since 2000, even in light of the genocide in the 1990s. This was in large part due to a clear national vision and health priorities, effected through strong coordination of international resources and the stakeholders behind them. Rwanda was exceptional at holding donor organisations to a common, nationally led strategic plan, and ensuring that spending was aligned with the national vision for the health sector. That vision, detailed in the national government’s Vision 2020 document (released in 2000), encouraged investment into ‘horizontal’ health system improvement and ensured more comprehensive care for mothers and children. Between 2000 and 2017, Rwanda achieved a 70% reduction in under-five mortality, nearly the highest reduction in the world.^9^

Peru cut its stunting rate by more than half in less than 10 years, in part by increasing demand. It did so despite a deadly civil war that claimed an estimated 70 000 lives during the 1980s and 1990s.^23^ Peru’s subsequent health strategies focused on a multisectoral, data-driven set of interventions that identified and targeted the country’s poorest, especially those living in remote and food insecure settings, with a specific focus on maternal nutrition and access to care.^24^ Starting in 2002 (and notably expanded in 2007), Peru established a free health insurance programme for low-income pregnant women and young children, known as *Seguro Integral de Salud*. Together with a nationwide conditional cash transfer programme known as *Juntos*, and a results-based financing programme known as *Programa Presupuestal Salud Materno Neonatal*, health service utilisation doubled,^25^ and stunting rates decreased from 28% to 13% between 2008 and 2016.^10^

Bangladesh found outstanding success in child mortality through multiple contributing factors, including interventions that were specific to social determinants of health. Through the 1990s and early 2000s reports of severe and widening socioeconomic disparities in Bangladesh notably highlighted inequalities in health services.^26^ Assisted by a strong non-governmental organisation sector and local research organisations capable of influencing policy-making, Bangladesh has made great strides in women’s empowerment since then. Microfinance programmes, introduced as far back as the 1980s, were found to improve women’s physical mobility, social connections and empowerment in household decision-making.^27^ This in turn enabled women to act on health information from CHWs, thus enhancing the effectiveness of health education campaigns.^28^ Family planning and education policies also empowered women and narrowed gender disparities. For example, the Female Secondary School Stipend Project provided payments for girls who attended and succeeded in school. Women’s literacy rates also increased from 41% to 62% between 2001 and 2015,^29^ and women’s workforce participation increased from 26% to 36% between 2003 and 2016. There were also reductions in total fertility rates and increases in birth spacings.^30^ Greater women’s empowerment, a success of its own, was among the many factors that helped Bangladesh reduce child mortality rates by 61% from 2000 to 2017.^9^

## Conclusion - Exemplars in Global Health: a road map

These lessons matter now more than ever. In times of relative stability, strategies and success stories offer important information for planning and continuous improvement. But in the face of alarming forecasts of the indirect effects of COVID-19 on maternal and child health, countries are in especially grave need of strategies that have demonstrably worked in the past and done so in complex—sometimes emergency—settings. These strategies can help prevent, mitigate or respond to the indirect impact of COVID-19 on nutrition and maternal and child health. The best emergency response systems may not be emergency systems per se, but everyday systems that have been employed successfully and can be amplified during a crisis.

While it is, in some cases, too early to know whether these keys to success are directly resulting in a successful response to the COVID-19 pandemic, the case for learning from their stories is substantiated by their impressive achievements in the past.

Over the past 3 years, we have incubated a new programme called Exemplars in Global Health (EGH)^31^ for the purpose of learning from positive outliers (deemed ‘Exemplars’) such as those highlighted here. EGH is a partnership that brings together local experts, funders and international collaborators with the mission of identifying positive global health outliers, understanding what makes them successful and disseminating the core learnings so they can be replicated in comparable settings. EGH continues to uncover further lessons, including from Exemplars in child mortality, stunting, community health, and soon epidemic preparedness and response, vaccine delivery, maternal mortality, neonatal mortality and primary healthcare. An understanding of the paths Exemplar countries took to achieve their successes may be most useful now to help avert indirect consequences of COVID-19, by highlighting what solutions may work, and how to deliver them.
